# The Many Faces of Innate Immunity in SARS-CoV-2 Infection

**DOI:** 10.3390/vaccines9060596

**Published:** 2021-06-04

**Authors:** Nicholas Hanan, Ronnie L. Doud, In-Woo Park, Harlan P. Jones, Stephen O. Mathew

**Affiliations:** 1 Graduate School of Biomedical Sciences, University of North Texas Health Science Center, Fort Worth, TX 76107, USA; NicholasHanan@my.unthsc.edu (N.H.); RonnieDoud@my.unthsc.edu (R.L.D.J.); Inwoo.Park@unthsc.edu (I.-W.P.); Harlan.Jones@unthsc.edu (H.P.J.); 2 Department of Microbiology, Immunology and Genetics, University of North Texas Health Science Center, Fort Worth, TX 76107, USA

**Keywords:** SARS-CoV-2, COVID-19, innate immunity, cytokines

## Abstract

The innate immune system is important for initial antiviral response. SARS-CoV-2 can result in overactivity or suppression of the innate immune system. A dysregulated immune response is associated with poor outcomes; with patients having significant Neutrophil-to-Lymphocyte ratios (NLR) due to neutrophilia alongside lymphopenia. Elevated interleukin (IL)-6 and IL-8 leads to overactivity and is a prominent feature of severe COVID-19 patients. IL-6 can result in lymphopenia; where COVID-19 patients typically have significantly altered lymphocyte subsets. IL-8 attracts neutrophils; which may play a significant role in lung tissue damage with the formation of neutrophil extracellular traps leading to cytokine storm or acute respiratory distress syndrome. Several factors like pre-existing co-morbidities, genetic risks, viral pathogenicity, and therapeutic efficacy act as important modifiers of SARS-CoV-2 risks for disease through an interplay with innate host inflammatory responses. In this review, we discuss the role of the innate immune system at play with other important modifiers in SARS-CoV-2 infection.

## 1. Introduction

Pandemics are a serious global health concern that demonstrate how interconnected the world has become. Severe acute respiratory syndrome coronavirus 2 (SARS-CoV-2), a novel coronavirus (CoV) capable of human infection, emerged from a seafood market in Wuhan, China, in December 2019 [1]. Coronaviruses are enveloped, positive-sense, and single-stranded RNA viruses known to cause disease in mammals and birds, with human endemic strains causing minor upper respiratory infection in immunocompetent individuals [2,3]. Similar to its novel CoV epidemic predecessors, the severe acute respiratory syndrome coronavirus (SARS-CoV) and the Middle East respiratory syndrome coronavirus (MERS-CoV), SARS-CoV-2 is in the genus betacoronavirus and is of zoonotic origin, which frequently causes upper and lower respiratory infection with potentially life-threatening complications [2,3,4,5]. Critical patients can develop acute respiratory distress syndrome (ARDS), a hyperinflammatory lung disease, and require prolonged mechanical ventilation and intensive care.

SARS-CoV-2 was declared a pandemic by the World Health Organization (WHO) on 11 March 2020 after it had spread throughout the world, transmitted by respiratory droplets and person-to-person contact [1]. As of 2 June 2021, over 170 million confirmed cases and over 3.55 million confirmed deaths have been reported worldwide [1]. Viral infection with SARS-CoV-2 was named coronavirus disease 2019 (COVID-19) by WHO to distinguish it from the related virus and disease responsible for the 2002–2003 outbreak with a similar name [1]. SARS-CoV-2 shows 79.6% sequence homology with the 2002 epidemic virus SARS-CoV, sharing the cell entry mechanism by an angiotensin converting enzyme II (ACE2) receptor [4]. COVID-19 is less lethal than SARS-CoV or MERS-CoV based on case fatality rates but more communicable overall, and thus exceeded these CoV in both total cases and deaths in a relatively short period [6]. Patients with SARS who recovered later showed some degree of pulmonary fibrosis [7], which causes concern for the potential long-term impact of COVID-19.

The vast majority of confirmed patients in China with COVID-19 experienced mild disease with symptoms of fever, dry cough, and fatigue, but 14% of cases were classified as severe with symptoms of dyspnea and low blood oxygen saturation, and 5% as critical with symptoms of respiratory failure, septic shock, or multiple organ failure (5). The case fatality rate was 2.3% for all COVID-19 patients, but death occurred only in critical cases with a subsequent fatality rate of 49% [5]. Deaths were associated with older age and pre-existing comorbidities [5], both of which contribute to an overall weaker immune response.

Currently there exists no definitive therapeutic treatment for COVID-19 and care is largely supportive and experimental while relying on the patient’s own immune system to clear the virus. A crucial component of an effective antiviral immune response is in the innate immune system, which can hinder viral replication while removing infected cells during the onset of disease and potentially resolve an infection during an early phase with a robust effort. The innate immune system is an essential initial response to infection by a pathogen, and patients whose systems fail to respond efficiently during the onset may have problems containing it later.

**Figure 1:**
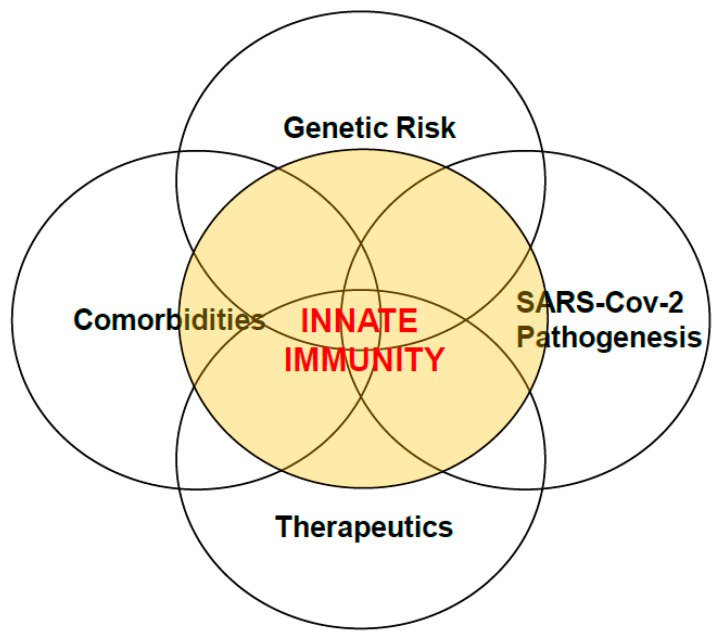
Integrated framework of important modifiers of SARS-CoV-2 risks for disease intersecting with innate immunity. Genetic risks, pre-existing co-morbidities, viral pathogenicity, and therapeutic efficacy are vital modifiers of SARS-CoV-2 risks for disease through an interplay with innate host inflammatory responses.

The complexities regulating immune competency is vast, consisting of individual and environmental inputs. Here, we present an integrated framework (for example, genetic risks, pre-existing co-morbidities, viral pathogenicity, and therapeutic efficacy) at play as important modifiers of SARS-CoV-2 risks for disease through an interplay with innate host inflammatory responses (Figure 1). Specifically, an understanding of these and other complex relationships will provide important insight into how alterations in the innate immune response in severe COVID-19 caused by SARS-CoV-2 would inform new therapies and treatments that mitigate viral replication as well as overactive inflammation created by the innate immune systems.

## 2. SARS-CoV-2 and COVID-19 Pathogenesis

Severe acute respiratory syndrome coronavirus 2 (SARS-CoV-2), a causative agent of coronavirus disease 2019 (COVID-19), the severe respiratory illness that is now rampaging the world, is an enveloped, single positive-strand RNA virus [8]. The envelope consists of a lipid bilayer derived from the cell membrane of the host and four structural proteins, spike (S), envelope (E), membrane (M), and nucleoprotein (N), as well as variable number of nonstructural proteins.

The most common route of transmission of SARS-CoV-2 is via infectious respiratory droplets and person-to-person contact. Likely portals of infection include the conjunctival epithelium, the nasal epithelium, and inhalation via the mouth. High viral load during the initial infection is an important predictive factor for the severity of the disease. Patients who experience repeated exposures, such as healthcare workers, are more likely to develop severe disease [9]. SARS-CoV-2 replicates extensively in the bronchial epithelium, which could help explain the high levels of transmission [10].

SARS-CoV-2 primarily enters human cells through the angiotensin-converting enzyme 2 (ACE2) receptor although other routes of entry of the virus cannot be excluded [11,12,13]. As the primary receptor, ACE2, which is a carboxypeptidase, acts within the Renin-Angiotensin-Aldosterone system (RAAS) and catalyzes the conversion of angiotensin II into angiotensin 1–7. ACE2 counteracts the effects of angiotensin II and helps regulate vascular tone and importantly for SARS-CoV-2, alveolar secretion of angiotensin II in the lung [14]. ACE2 is expressed in many different cell types, including the nasal and alveolar epithelium, the heart, the blood vessels, and the kidneys [15,16]. The SARS-CoV-2 spike protein is estimated to have approximately 10 to 20 times greater affinity for the ACE2 receptor than that of SARS-CoV [17]. ACE2 expression in the nasal epithelium and the lungs generally increases with age, which may help explain why adults and elderly patients are more susceptible to infection [18]. Studies have also shown that CD209L and CD209 serve as alternative receptors for SARS-CoV-2 in disease-relevant cell types, including the vascular system [19]. SARS-Cov-2 has a furin cleavage site (PRRAR) in its spike protein that is absent in other group-2B coronaviruses, which plays a critical role in infection and pathogenesis [20]. Daly et al. showed that a component of SARS-CoV-2 S protein binds to cell surface neuropilins (NRP1 and NRP2) via the S1 CendR motif generated by the furin cleavage of S1/S2, which could be a potential therapeutic target [21].

Coronavirus cellular entry is dependent on binding of the spike protein (S) to a specific cellular receptor, followed by S priming by cellular proteases [15,22]. A cysteine protease that is important for SARS-CoV-2 entry is TMPRSS2, which is also expressed in the nasal and bronchial epithelium [15,23,24,25,26]. However, TMPRSS2 is not found in all ACE2 positive cells, suggesting that SARS-CoV-2 might use alternative pathways. SARS-CoV-2 can enter TMPRSS2 negative cells via cathepsin B/L7 [22,23]. Cathepsin B is more widely expressed than TMPRSS2 in ACE2 positive cells, being found in 70–90% of ACE2 positive cells. While TMPRSS2 activity is documented to be important for viral transmission [27,28], the potential of cathepsin B/L or other proteases to functionally replace TMPRSS2 has not been determined [15]. Mature enterocytes that express high levels of ACE2 receptor were found to be susceptible to SARS-CoV-2 infection with TMPRSS2 and TMPRSS4 enhancing the viral entry into the enterocytes [29].

Upon entry, the infected SARS-CoV-2 is known to encode a polyprotein proteolytically processed into 16 nonstructural proteins (Nsp1–16) from open reading frame (Orf) 1a/b, structural proteins including S, E, M, and N, and 9 accessory proteins from Orf3a, 3b, 6, 7a, 7b, 8, 9b, 9c and 10 [30,31]. Viral gene and the expressed proteins in the infected cells then trigger the host immune responses, and the innate immune cells initiate an inflammatory cascade. However, the signaling mechanisms responsible for induction of inflammatory cytokines by SARS-CoV-2 has not been fully elucidated.

## 3. Innate Immune Cells & Their Role in COVID-19

### 3.1. Overview of the Interplay between the Innate Immune Responses and SARS-CoV-2

Many components of the innate immune system are important for initial detection and clearance of viral infections. The key pathogenic features of COVID-19 different from the diseases caused by other coronaviruses SARS-CoV and MERS-CoV is that the mortality rate for SARS-CoV-2 is relatively reduced, while its interpersonal transmissibility is comparably elevated. Another notable clinical feature is that the severity of the disease among infected patients range from asymptomatic to symptomatic, and even among the symptomatic patients, approximately 80% of the infected patients show mild symptoms, whereas 15% of the confirmed cases progress to the severe phase. Finally, as noted previously, the elderly and those with co-morbidities, such as diabetes, obesity and cardiovascular, respiratory, renal, and lung diseases, are the most susceptible to COVID-19 and its severe disease complications. These clinically distinctive features point toward differential virus/immune responses that vary with the particular interplay between the viral species and the given patient’s host cells. Lucid understanding of the clinical immune response variances for SARS-CoV-2 is therefore imperative in combating COVID-19.

The entry of SARS-CoV-2 is initiated by the interaction between the viral spike protein, S, and its cognate receptor molecule, ACE2, expressed on the surface of the respiratory epithelial cells of the host [32], wherein the host serine protease, TMPRSS2, which cleaves viral spike protein at the S1/S2, plays an important role for the virus entry [33]. Upon virus entry into the cell cytoplasm via endocytosis, antiviral innate immune signaling pathways in the virus-infected host are activated to thwart virus replication [34]. Innate immune responses are activated by recognition of pathogen-associated molecular patterns (PAMPs; the conserved components of microbes) through a large repertoire of pattern-recognition receptors (PRRs) [35,36]. The most well-characterized PRRs in sensing different types of PAMPs are toll-like receptors (TLRs), retinoic acid-inducible gene I (RIG-I)-like receptors (RLR), NOD-like receptors, and C-type lectin receptors (CLRs) [34], and recognition of the PAMPs by these PRRs activates their specific subsequent signaling pathways to induce synthesis of various antiviral molecules, such as interferons (IFNs) and pro-inflammatory cytokines [37,38,39,40]. Cytokines secreted by the activated innate immune system then stimulate the adaptive immune responses, recruit various immune cells to the site of infection, and help to inhibit viral replication. Granulocytes release enzymes and toxic proteins in an effort to kill viruses. Monocytes travel to sites of infection and differentiate into monocyte-derived macrophages and dendritic cells. These macrophages, along with neutrophils, phagocytose infected cells and pathogens. Activated dendritic cells then take up the pathogen-derived antigens and present them to naive T-helper cells to stimulate the adaptive immune response. The complement system also participates in immune cell recruitment, activation, and ultimately destruction of pathogens.

Type I interferons are among the first cytokines to be upregulated in virus-infected cells and are important in coordinating the antiviral response and inflammation [41]. However, the IFN system is generally significantly suppressed in most severe SARS-CoV-2 patients [42]. Schroeder et al. showed that SARS-CoV-2 suppresses cytokine induction and interferon signaling with lower efficiency than SARS-CoV, despite the shared genome architecture and expression of homologous viral proteins [43]. Studies have shown that SARS-CoV-2 proteins antagonize type I interferon production and signaling [44,45]. Whole exome or genome sequencing in patients with life-threatening COVID-19 pneumonia revealed that inborn errors of TLR3- and IRF7-dependent type I IFN immunity could underlie life-threatening COVID-19 pneumonia in patients with no prior severe infection [46]. All of these studies highlight the important role of Type I interferons in SARS-CoV-2 infections and Type I IFN administration may be of therapeutic benefit in selected patients, at least early in the course of SARS-CoV-2 infection.

### 3.2. Overactivity of the Innate Immune System

In spite of these critical antiviral functions, an overactive innate immune response can contribute to disease pathogenesis [47]. Several previous studies have reported an association between dysregulated secretion of cytokines and progression to severe SARS-CoV-2 [48,49]. Patients with severe and critical cases of COVID-19 often have high levels of cytokines including IL-1, IL-2, IL-6, IL-7, IL-10, G-CSF, IP-10, MCP-1, MIP-1α, and tumor necrosis factor-α (TNFα) [47]. The overabundance of these cytokines can lead to a “cytokine storm”, which may play a large role in COVID-19 pathogenesis and initiation of viral sepsis and inflammatory-induced lung injury. These patients often develop viral pneumonia, which may progress to acute respiratory distress syndrome (ARDS), or even multi-organ failure [10,41,50,51].

### 3.3. Cytokines, Chemokines, and Hyperinflammation

Significant increases in inflammatory cytokines and chemokines in serum levels were seen for severe COVID-19 patients, but it is currently uncertain if it is directly involved in further lung damage [52]. Different analyses have provided a wide array of various elevated cytokines that may potentially lead to local lung inflammation and disease progression. One study demonstrated significantly higher cytokine and chemokine plasma levels in COVID-19 patients, but no significant difference was seen when comparing mild and severe patients [53]. A correlation was shown in another study between increasing disease severity and circulating proinflammatory cytokines, including TNF-α, interleukin-2R (IL-2R), and interleukin-6 (IL-6) [52]. Another found elevated levels of IL-6, IL-1α, CCL2, CCL8, CXCL2, CXCL8, CXCL9, and CXCL16 but low levels of Type I and Type III interferons (IFN) [48].

In a comparison of bronchoalveolar fluid (BALF) samples for moderate and severe patients, severe patients showed increased levels of IL-6, IL-8, and IL-1β and chemokines CCL2, CCL3, CCL4, CCL7, CCL8, CXCL2, CXCL8, CXCL9, CXCL10, CXCL11, CXCL16, FGF, CSF-3, CSF-2, PDGF, and VEGF were also elevated [49,54,55]. Another BALF analysis found increased CCL2, CCL8, CXCL2, CXCL1, IL-33, and CCL3L1 and correlated disease severity of COVID-19 with the production of a cytokine storm [56]. Chemokines CCL2, CCL8, and CXCL10 chemically attract monocytes to a location, while CXCL2 and CXCL8 (also called IL-8) is a chemotactic for neutrophils and T cells [54,56]. Recruitment of leukocytes to the lungs is necessary for viral clearance but excessive inflammation can result in fibrosis, poor alveolar gas exchange, and patient deterioration. Lung interstitial tissue showed extensive infiltration from CD4+ T cell, CD8+ T cell, macrophages, and GZMB+ cells [53].

**Figure 2:**
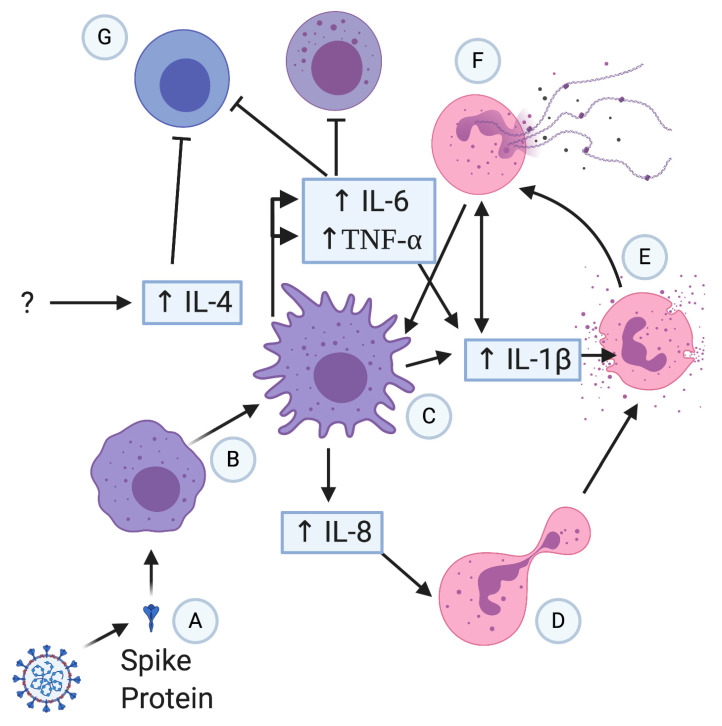
Potential severe patient hyperinflammation progression in COVID-19. Spike protein (**A**) activates macrophages (**B**) that release various proinflammatory cytokines; (**C**) IL-8 attracts neutrophils (**D**), which have the potential to degranulate (**E**) and form NETs (**F**). NETs can potentially form a positive feedback loop with macrophages and IL-1β release. Macrophages release IL-6 and TNF that inhibits and causes apoptosis in T cells and NK cells (**G**), causing or exacerbating lymphopenia.

Neutrophils have been hypothesized to participate in a COVID-19 induced cytokine storm and development of ARDS due to evidence of infiltration into the lungs seen in autopsies, including pulmonary capillaries and the alveolar space [57]. Through the formation of neutrophil extracellular traps (NETs) as shown in Figure 2, they can induce IL-1β expression from macrophages which has been shown to cyclically induce further NET formation in some disease states [57]. NETs are extracellular structures made from the neutrophil’s DNA and microbicidal proteins [58], which function to entangle pathogens and have been implicated in exacerbating pulmonary diseases, including ARDS [57]. Blood serum of COVID-19 patients displayed elevated markers indicating NET formation, including citrullinated histone H3 (Cit-H3) and myeloperoxidase-DNA (MPO-DNA), which also was further shown to have activated NET formation in control neutrophils when the COVID-19 sera was applied in vitro [58]. When compared to hospitalized COVID-19 patients breathing room air, COVID-19 patients on mechanical ventilation had significantly higher levels of circulating cell-free DNA and MPO-DNA but not Cit-H3 or neutrophilia [58]. Circulating cell-free DNA and MPO-DNA were positively correlated with absolute neutrophil count [58]. In contrast, deoxyribonuclease I (DNase I) can work to promote the clearance of overproduced NETs and, thus, minimize unwarranted neutrophil-mediated collateral damage [59]. Immunomodulation through activation of the innate immune sensor toll-like receptor 5 (TLR5) that recognizes flagellin could potentially act as a trojan horse “danger” signal, which may trick the host into thinking that immune responses are required to suppress a “bacterial” infection but instead activates antiviral responses to eliminate SARS-CoV-2 [60].

COVID-19 induced sepsis was categorized by Giamarellos-Bourboulis et al. into three classifications: macrophage activation syndrome (MAS), immunoparalysis with downregulation of human leukocyte antigen D related (HLA-DR) on CD14 monocytes, and an intermediate state without apparent immune dysregulation [61]. Patients with severe respiratory failure (SRF) exhibited either MAS, which is associated with elevated levels of IL-1β, or immune dysregulation, which is associated with IL-6 [61]. Approximately, a fourth of COVID-19 patients with SRF had indications of MAS, while the majority had immune dysregulation [61]. Immune dysregulation was correlated with increased neutrophil and monocyte counts, as well as increased IL-6 and C reactive protein [61]. Low HLA-DR is caused by monocyte hyperactivation, high levels of circulating IL-6, and lymphopenia [61]. All of these innate immune responses lead to a severe hyperinflammation in COVID-19 patients (Figure 2).

To date there has been little evidence of direct infection of monocytes or macrophages by SARS-CoV-2, but studies have shown that SARS-CoV and MERS-CoV infection results in aberrant cytokine production [62]. Only MERS-CoV was capable of active viral replication inside monocyte-derived macrophages (MDM), but both viruses failed to activate antiviral cytokines IFN-α and IFN-β within infected MDM while significantly increasing TNF-α and IL-6 expression [62]. Another study confirmed severe COVID-19 patients correlated with increased TNF-α as well as IL-4, where TNF-α was associated with lymphocyte apoptosis and IL-4 interfered with T regulatory cell activity [63]. A murine animal model of infected macrophages with the spike (S) protein from SARS-CoV demonstrated the upregulation of TNF-α and IL-6 caused by inducing the NF-κB pathway [64]. This was later confirmed with human peripheral blood monocyte macrophages, where the S protein activated monocytes increased TNF-α and IL-6 along with a significant increase in IL-8 that was dose dependent [65].

Autopsy examination of six COVID-19 patients revealed SARS-CoV-2 infection of the spleen and lymph nodes with extensive damage to its tissues as well as follicle depletion [66]. Viral proteins were found in the spleen and lymph node resident CD169+ macrophages, but not in CD3+ T cells or B220+ B cells, indicating damage was likely macrophage related and SARS-CoV-2 potentially migrated to the locations utilizing infected macrophages [66]. Infected macrophages were reported to have also increased expression of IL-6, a cytokine known to induce apoptosis in lymphocytes and potentially a cause of lymphopenia in COVID-19 patients [66].

### 3.4. Lymphopenia

Lymphopenia may contribute to overall disease progression. SARS-CoV was known to cause lymphopenia in SARS patients with altered lymphocyte subsets [67], but whether that was the result of the viral infection or due to following treatment for inflammation with glucocorticoid steroids has remained uncertain [68]. COVID-19 appears to share this characteristic lymphopenia [53,69]. The exact mechanism is currently unknown for what directly causes the depletion of lymphocytes and resulting lymphopenia in COVID-19 patients. Lymphocytes express the ACE2 receptor utilized by SARS-CoV-2 for infection [69] but there is no evidence of actual viral infection of the lymphocyte demonstrated from viral gene expression within the cell [56]. Peripheral blood mononuclear cells (PBMC) have been shown to have increased expression of p53 signaling pathways and cellular apoptosis, which could potentially have been induced from SARS-CoV-2 [56].

In retrospective studies, a correlation between decreasing lymphocyte subset levels and increasing severity of COVID-19 patients has been demonstrated [52,63,69], including the suggestion by Tan et al. to use lymphopenia as a predictive indicator of disease severity and patient prognosis [69]. Patients with onset of symptoms and blood lymphocyte levels over 20% on hospitalization or which subsequently increased to over 20% following treatment had positive outcomes, but below 5% were deemed critically ill with corresponding high mortality [69]. Lymphocyte subsets in other studies further showed a decreased absolute T cell, CD4 T cell, cytotoxic (CD8+) T cell, and natural killer (NK) cell count, but with a lower normal range value for B cells [52]. T cell counts decreased significantly in COVID-19 cases of non-severe and severe patients, with severe patients having drastically reduced levels [52,63]. Song et al. reported an overall decreased lymphocyte subset including CD3+ T Cells, CD4+ T Cells, CD8+ T cells, and NK cells but with no significant difference between mild and severe patients, except for B cells, which were increased in severe patients [53].

Severe patients also had neutrophilia alongside lymphopenia, creating higher values in the neutrophil-to-lymphocyte ratio (NLR), which serves as a potential biomarker for increased systemic inflammation [52]. Increased NLR was associated with lung inflammation and the eventual development of critical patients with ARDS leading to an ultimately poor prognosis, and is another prospective marker for differentiating COVID-19 patients by severity [70,71]. Values above 3.3 of NLR for patients over 50 years of age showed a predictive deteriorative change from mild to severe disease at an average of approximately six days [71].

Cytotoxic T cells and natural killer cells are essential for an effective antiviral immune response. They were shown to be functionally exhausted in severe patients by the increased expression of NKG2A on lymphocytes, an inhibitory immune receptor on the cell surface which decreases its activity and may be correlated with disease progression [72]. NKG2A was shown to be upregulated in severe COVID-19 patients by Zheng et al., alongside decreased functional activity indicated from the decrease of intracellular cytokines CD107a, interferon-γ (IFN-γ), IL-2, and TNF-α [72]. Membrane protein CD107a is correlated with NK cell activity and further cytokine secretion, with decreased function suggesting potential excessive lung inflammation is not due to overactivation of NK cells [73]. Patients who successfully recovered displayed subsequent lower NKG2A levels that had returned to baseline, while critical cases displayed a hindered antiviral immune response early in the disease progression [72].

Further analysis by Demaria et al. confirmed lymphopenia and increased NKG2A expression in severe COVID-19 patients with pneumonia and ARDS, but also found a decrease in mature NK cells in patients with ARDS in both the peripheral blood and the bronchoalveolar fluid (BALF), indicating systemic deficiency instead of cell migration [74]. They examined NK cells for the presence of immunosuppressant receptors NKG2A and PD-1 and the enzyme CD39 for a potential immunotherapy avenue of treatment, and found all to have been upregulated in the BALF of ARDS patients with a higher degree of overall expression than seen in the periphery [74]. Subsequent treatment in blocking NKG2A activation with the use of the monoclonal antibody monalizumab restored NK cytotoxic function, and could potentially recover NK antiviral activity in patients [74].

### 3.5. Immunosuppression

Patients with respiratory failure from severe SARS-CoV-2 commonly display one of two major forms of SARS-CoV-2 immune dysregulation: the “cytokine storm” phenotype, or more commonly, one characterized by targeted immunosuppression. Patients with the immunosuppression phenotype have elevated levels of IL-6 and IL-8, relatively lower levels of cytokines in other pathways, and the virtual absence of a type I or type II IFN response [42]. In patients with this phenotype, monocytes are generally less activated and lymphocytes, with the exception of plasmablasts, are noted to be fewer in number with several subsets showing signatures of suppression such as the reduced type I and type II IFN signaling. One possible cause for this immunosuppression is excessive glucocorticoid levels, which may cause systemic inflammation, which is known to suppress HLA-DR expression on monocytes [75]. The elevated levels of IL-6 can increase cortisol levels through various mechanisms, including direct stimulation of the adrenal cortex and through induction of corticotropin-releasing hormone and adrenocorticotropin [42].

## 4. Co-Morbid Conditions and Genetic Risks Stoke Immunopathology of SARS-CoV-2

### 4.1. Co-Morbidities: A Target for SARS-CoV-2

Pre-existing health risks demonstrate poorer clinical outcomes from SARS-CoV-2 [15,16]. In a recent review by Phillips et al. and others, the incidence and morbidity associated with SARS-CoV-2 was highest among individuals with underlying chronic conditions including obesity, cardiovascular, hypertension, kidney, type 2 diabetes mellitus, and respiratory-related disease [76,77,78,79,80]. ACE2, which plays a critical role in the regulation of both underlying disease and SARS-CoV-2 entry, is essential for metabolic control of respiratory, vascular, myocardial, kidney, and pancreatic functioning, among others [81,82,83]. For example, in the case of metabolic disease, an upregulation of ACE2 that interacts with the Angiotensin-II type 1 receptor (AT1R) has pathologic pro-inflammatory and pro-fibrotic effects [84,85]. Previous studies have shown that entry and replication of SARS-CoV-2 through ACE2 receptors triggers release of large numbers of cytokines, especially IL-6, causing acute fatal “cytokine storms” provoking SARS-CoV-2 pathobiology.

ACE2 is highly expressed in alveolar epithelial cells, which facilitates SARS-CoV-2 infection in the lung. Despite the ACE2 receptor serving as a point of entry, ACE2 has a protective effect on the lungs. It regulates angiotensin II, of which high levels can cause increased vascular permeability and lead to lung, cardiac, and vascular damage. As SARS-CoV-2 progresses, it activates immune cells, platelets, and coagulation factors, which can cause multiple organ failure and death. Importantly, SARS-CoV-2 downregulates levels of ACE2, which may disrupt the balance of angiotensin II and play an important role in disease pathogenesis [86,87]. In a phase II trial, patients who received recombinant ACE2 saw a reduction in angiotensin II levels, which appeared to improve lung injury [14,88,89]. This suggests that drugs such as ibuprofen that raise ACE2 levels may be beneficial in patients with severe SARS-CoV-2 who have sustained lung injury. Recently, the process of inflammaging has received much attention which could explain some of the pathology that is seen in elderly patients with SARS-CoV-2 infection as the lungs of elderly individuals are characterized by chronic low-grade inflammation [90].

There have also been instances of acute cardiac inflammation and injury in convalescent COVID-19 patients. Regardless of preexisting conditions and infection severity, 78% of patients in a prospective cohort study who had recovered from COVID-19 showed some degree of cardiac involvement on cardiac magnetic resonance imaging [91]. The most common of which being myocardial inflammation, followed by regional myocardial scar and pericardial enhancement [91]. These existed despite overall COVID-19 severity and continued after the acute infection phase, hinting at a potential long-term toll the virus can take on the body [91]. Severe pediatric COVID-19 patients without respiratory failure or comorbidity in Paris were reported to have myocarditis, diffuse inflammation, and an atypical Kawasaki disease, further indicating that SARS-CoV-2 or its induced systemic inflammation may have considerable effect on the cardiovascular system [92].

### 4.2. Genetic-Associated Risks: Influences of Age, Race, and Gender Disparities

SARS-CoV-2 shows significant age disparities. In general, the susceptibility to and severity of disease tends to increase with age. Fewer children contract SARS-CoV-2, and of those infected, the disease is generally less severe. Elderly patients suffer from much higher susceptibility and mortality than children do. A prominent factor regarding infection rate in children may be the early closing of schools and daycares, therefore reducing exposure [93]. Other factors may include trained immunity, fewer comorbid risk factors, and the fact that children’s lungs are still developing. Trained immunity is functional reprogramming of cells of the innate immune system to a more active state after stimulation by certain antigens, such as those from vaccinations or viral infections. This occurs through metabolic reprogramming, such as enhancement of the Krebs cycle, and epigenetic changes, such as acetylation or demethylation, resulting in enhanced transcription of IL-1β, IL-6 and TNF-α genes [93]. These changes can affect local cells, such as lung macrophages and dendritic cells, as well as progenitor cells of monocyte and myeloid cell lines. It also activates NK cells and interferons, leading to strong innate immune responses that may help contain early infections and clear them more rapidly.

Children also less commonly have risk factors such as obesity, smoking, and comorbidities such as hypertension and diabetes mellitus. However, children who have these pre-existing factors or illnesses may still fall into high-risk categories and need appropriate monitoring. Additionally, since children’s lungs are still developing, the alveolar epithelium have a greater capacity to regenerate, which may accelerate recovery (31). In contrast, adult patients have weaker adaptive immunity and in many SARS-CoV-2 cases, dysfunctional hyperactive innate immune responses in severe infections that is not commonly observed in children [93]. This is especially common in the elderly population, who have a much higher mortality rate from SARS-CoV-2.

The majority of SARS-CoV-2 cases are among non-Hispanic whites, but racial and ethnic minorities are disproportionately represented. Ethnic minorities especially African American (AA) populations may have a greater risk of SARS-CoV-2 infections due to comorbidities like hypertension, cardiovascular disease, diabetes, chronic obstructive pulmonary disease (COPD), and asthma [94,95,96]. In addition, it was observed that AAs and Hispanics/Latinos when compared with non-Hispanic Whites had higher mortality associated with COVID-19 [96]. Social determinants of health that may contribute to these disparities include housing, neighborhood and environment, and education and wealth gaps [97]. Racial and ethnic minorities are more likely to live in areas with higher rates of acute SARS-CoV-2 infections. Additionally, they are more commonly subjected to crowded living conditions and reliance on public transportation. Minorities are also disproportionally represented as essential workers at jobs such as grocery stores, food services, warehouses, factories, and healthcare facilities, increasing their risk of exposure [98]. They also generally have lesser access to testing for SARS-CoV-2, and more commonly lack health insurance if they do become sick.

SARS-CoV-2 also shows significant gender disparities. Although men and women have a near-equal case rate, the mortality rate was higher in males [10,99]. Male patients generally have greater inflammation indices, more impaired liver and kidney function, and more complications than women do. They also generally have more significant lymphopenia and thrombocytopenia [100]. Evidence suggests that more men die than women, which may be due to sex-based immunological differences [101,102], including genetic differences in chromosome complement and different levels of sex hormones [103]. Estrogens serve to stimulate the humoral response to viral infection, while testosterone and progesterone act to suppress both innate and cell-mediated immune responses. It is possible that administration of these hormones, such as via oral contraceptive, could keep estrogen levels high and therefore play a protective role [10]. This protective role likely outweighs the risk of thrombotic events [10,104]. Another possible factor is the role of smoking in aggravating the disease, which is more common in men [10].

## 5. Ongoing Therapeutic Approaches

There are currently 5503+ total clinical trials ongoing worldwide regarding COVID-19 (Clinicaltrials.gov). Several antiviral drugs activate the innate immune system against SARS-CoV-2. Remdesivir, a nucleotide prodrug of an adenoside analog, is the only drug that is currently approved by the FDA for the treatment of SARS-CoV-2. Remdesivir has demonstrated in vitro activity [105] against SARS-CoV-2 and inhibits viral replication by binding to the viral RNA-dependent RNA polymerase, therefore causing premature termination of RNA transcription. Administration of remdesivir is recommended for hospitalized patients who require supplemental oxygen, but not for mechanically ventilated patients, as there is no data that shows a benefit at this advanced disease stage.

The corticosteroid dexamethasone is strongly recommended for all patients who require supplemental oxygen, with the greatest benefit occurring in patients who are mechanically ventilated [106]. While combination therapy of remdesivir and dexamethasone theoretically may be beneficial for patients with severe SARS-CoV-2, this has not been rigorously examined in clinical trials. However, combination therapy of dexamethasone with the IL-6 inhibitor Tocilizumab has been found to improve survival of patients exhibiting rapid respiratory decompensation due to SARS-CoV-2 [107].

Preliminary studies have shown that outpatients may benefit from receiving SARS-CoV-2 monoclonal antibodies early in the course of infection. The anti-SARS-CoV-2 monoclonal antibodies bamlanivimab and casirivimab plus imdevimab are available through Emergency Use Authorizations (EUA) for outpatients who are at high risk for disease progression. Currently due to insufficient data, NIH Panel does not recommend either for or against the use of these anti-SARS-CoV-2 monoclonal antibodies (bamlanivimab or casirivimab plus imdevimab) in nonhospitalized patients with mild to moderate COVID-19 (https://www.covid19treatmentguidelines.nih.gov/, accessed on 3 June 2021).

Among the vaccine candidates, FDA has issued an EUA for Moderna’s mRNA-1273 COVID-19 vaccine and Pfizer-BioNTech’s BNT162b2 COVID-19 mRNA vaccine and Johnson & Johnson’s adenovirus vaccine. Several other vaccine candidates are in phase 3 clinical trials and have received an EUA in several countries [108]. An interim analysis of four randomized controlled trials in Brazil, South Africa and the UK for a chimpanzee adenovirus vectored ChAdOx1 nCoV-19 vaccine (AZD1222) has shown to have an acceptable safety profile and found to be efficacious in symptomatic COVID-19 patients [109]. Neutralizing anti-spike antibodies were detected in all subjects following a second booster shot with humoral and cellular immune response [110]. The current mRNA vaccines have single stranded RNA with modified nucleotides to decrease the binding to toll like receptors (TLRs) and other immune sensors preventing excessive production of type 1 interferons and other inflammatory mediators [111].

Both Moderna’s mRNA-1273 COVID-19 vaccine and Pfizer-BioNTech’s COVID-19 vaccine use newly licensed mRNA technology delivered in a liquid nanoparticle (LNP) system [111]. The mRNA in each vaccine encodes for production of the S protein of SARS-CoV-2, the primary target for both endogenous neutralizing antibodies and therapeutic monoclonal antibodies [112]. These vaccines have been highly effective and result in significant neutralizing antibody titers and virus-specific T-cell responses, with phase III clinical trials showing 90–95% efficacy in protecting against SARS-CoV-2 [112].

A vaccine requires both the pathogen-specific immunogen and an adjuvant in order to stimulate adaptive immunity. The adjuvant stimulates the innate immune system, providing the required second signal for activation of T cells. The mRNA in mRNA vaccines can act as both the immunogen and the adjuvant due to the intrinsic immunostimulatory properties of RNA. A critical step in the innate immune system’s response to viruses is recognition of single and double-stranded RNA, which is detected by various endosomal and cytosolic innate sensors upon cellular entry. In the endosome, endosomal Toll-like receptors such as TLR3 and TLR7 bind single-stranded RNA (ssRNA). In the cytosol, components of inflammasomes such as MDA5, RIG-I, NOD2, and PKR bind to both ssRNA and double-stranded RNA (dsRNA) [111].

Binding of ssRNA or dsRNA results in cellular activation and production of type I IFN and other various inflammatory mediators [113]. Both the Pfizer and Moderna vaccines contain purified ssRNA with modified nucleotides that reduce binding to TLR and other immune sensors, which helps to limit excessive production of type I IFN, which has an inhibitory function on cellular translation. The LNP carrier has a protective function for the mRNA and targets delivery to lymphatics to promote protein translation in the lymph nodes. The LNP is taken up by dendritic cells (DCs) in the lymph node upon entry, which process the antigen and present it to T cells to activate the adaptive immune response.

The other approved vaccine formulations such as the AstraZeneca ChAdOx1 nCoV-19 utilize DNA delivered by a non-replicating recombinant adenovirus (AdV) system. The ChAdOx1 nCoV-19 is slightly less effective with an average efficacy of 71% [109], while the Russia-developed Gam-COVID-vac (Sputnik V) had an average efficacy of 91% [114]. The viral particle that encases the DNA serves as the adjuvant in these vaccines. After injection, the AdV particles target innate immune cells such as macrophages and DCs. Binding to various pattern-recognition receptors that recognize dsDNA, especially TLR9, stimulates the innate immune response and induces type I IFN secretion.

Both the mRNA and AdV based vaccines converge in intracellular production of the S protein and type I IFN secretion. Production of the S protein and other various innate immune responses prime CD4^+^ and CD8^+^ T cells to differentiate into memory and effector subsets, respectively. Vaccine-driven type I IFN production promotes differentiation of CD4^+^ and CD8^+^ T cells. CD4^+^ follicular T cells promote differentiation of B cells into antibody-secreting plasma cells, and CD8^+^ effector cells produce inflammatory and cytotoxic mediators [111].

Multiple studies have shown the impact of IL-6 in severe COVID-19 patients and lung inflammation [48,52,54] and its association with immune dysregulation in different states of COVID-19 sepsis [61]. Treatment with the IL-6 inhibitor Tocilizumab increased total lymphocyte counts of six patients within 24 h, although overall patient outcome was not stated [61]. To date, numerous clinical trials are exploring blocking the IL-6 pathway with different therapeutic agents.

## 6. Future Global Disease Surveillance

Environmental factors play a considerable role in zoonotic disease transmission as changes in climate and animal habitat force adaptive responses from animal populations that serve as potential viral reservoirs [115]. Mammals of the order Chiroptera, commonly called bats, are pervasive viral reservoirs, which have reacted to the loss of natural habitat by cohabitating around and with humans and domesticated animals, which increases the likelihood of viral spillover events from direct and indirect interactions [115,116,117]. Bats are natural reservoirs for zoonotic viruses with the highest fatality rates in humans, including the *Betacoronaviruses* SARS-CoV-1, SARS-CoV-2, and MERS-CoV, Ebola, and rabies in part due to their constitutive expression of the interferon pathway promoting viruses that can rapidly propagate [118].

Preventing future pandemics would benefit from actively monitoring likely hotspots in addition, extensive wildlife screening. The viruses SARS-COV-1 and SARS-CoV-2 had intermediate hosts in animal markets between bat and human transmission, likely because of the overall poor sanitary conditions of the open-air markets [119]. This would facilitate disease transmission among numerous diverse species primed for infection from stress, crowded, and unhygienic enclosures, and infrequent or nonexistent previous encounters in nature for past viral exposure [116,119]. Ending or severely regulating wet markets could have a potential positive impact on preventing novel virus emergence, as well as educating the public of the possible dangers of contact with wild animals such as bats [116]. Decreasing the frequency of wildlife contact with other animals and humans could potentially limit the spread of disease. Regular disease surveillance at these markets and other likely hotspots using whole-genome sequencing and metagenomics could also identify sick animals earlier before a spillover occurs or have the origin or intermediate host known early on to apprise governmental response, where metagenomics has been shown to be able to identify novel coronaviruses and genome sequencing could help direct vaccination efforts [120,121].

## 7. Conclusions

Although we now have a few SARS-CoV-2 vaccines that have been approved by the FDA via EUA, a range of other immune interventions that are being explored, as a means to boost protective innate immunity and reduce damaging inflammatory responses, would be crucial in the fight against COVID-19. These immunomodulatory approaches could be utilized in combination with the current standard of care, the antiviral remdesivir and corticosteroid dexamethasone. Despite the challenges posed by this novel and rapidly spreading viral infection, the response from the scientific community has been tremendous with the development of several vaccine candidates and treatment options within a short duration of time, which will serve as a template for future responses to pandemics.

## Data Availability

Not applicable.
